# Neuronal progenitor cells-based metabolomics study reveals dysregulated lipid metabolism and identifies putative biomarkers for CLN6 disease

**DOI:** 10.1038/s41598-023-45789-7

**Published:** 2023-10-29

**Authors:** Corina-Marcela Rus, Daniel L. Polla, Sebastiano Di Bucchianico, Steffen Fischer, Jörg Hartkamp, Guido Hartmann, Yunus Alpagu, Claudia Cozma, Ralf Zimmermann, Peter Bauer

**Affiliations:** 1 Centogene GmbH, Am Strande 7, 18057 Rostock, Germany; 2 Joint Mass Spectrometry Center, Chair of Analytical Chemistry, University of Rostock, Albert-Einstein Straße 27, 18059 Rostock, Germany; 3 Helmholtz Zentrum München, Ingolstädter Landstraße 1, 85764 Neuherberg, Germany; 4 Department Life, Light & Matter, University of Rostock, Albert-Einstein Straße 25, 18059 Rostock, Germany; 5 Department of Medicine, Clinic III, Hematology, Oncology, Palliative Medicine, Rostock University Medical Center, Ernst-Heydemann-Str. 6, 18057 Rostock, Germany

**Keywords:** Biochemistry, Biomarkers, Diseases

## Abstract

Neuronal ceroid lipofuscinosis 6 (CLN6) is a rare and fatal autosomal recessive disease primarily affecting the nervous system in children. It is caused by a pathogenic mutation in the *CLN6* gene for which no therapy is available. Employing an untargeted metabolomics approach, we analyzed the metabolic changes in CLN6 subjects to see if this system could potentially yield biomarkers for diagnosis and monitoring disease progression. Neuronal-like cells were derived from human fibroblast lines from CLN6-affected subjects (n = 3) and controls (wild type, n = 3). These were used to assess the potential of a neuronal-like cell-based metabolomics approach to identify CLN6 distinctive and specific biomarkers. The most impacted metabolic profile is associated with sphingolipids, glycerophospholipids metabolism, and calcium signaling. Over 2700 spectral features were screened, and fifteen metabolites were identified that differed significantly between both groups, including the sphingolipids C16 GlcCer, C24 GlcCer, C24:1 GlcCer and glycerophospholipids PG 40:6 and PG 40:7. Of note, these fifteen metabolites were downregulated in the CLN6 disease group. This study is the first to analyze the metabolome of neuronal-like cells with a pathogenic mutation in the *CLN6* gene and to provide insights into their metabolomic alterations. This could allow for the development of novel biomarkers for monitoring CLN6 disease.

## Introduction

Neuronal ceroid lipofuscinoses (NCLs) are among the most frequently encountered groups of rare, inherited neurodegenerative lysosomal storage disorders in children^1–3^. The worldwide prevalence of NCLs varies based on the region and the variant type. Their frequency is estimated at 0.01 to 9 per 100,000 live births^4,5^. NCLs are caused by mutations in one of the thirteen CLN genes (*CLN1-CLN8, CLN10-CLN14*)^6,7^ that impact different proteins, one of which is the transmembrane protein CLN6^6^. Neuronal ceroid lipofuscinosis—type 6 (CLN6) [OMIM# 601780] is an autosomal recessive disease caused by pathogenic mutations in the *CLN6* gene that encodes for the CLN6 protein, whose function is not yet fully understood^8,9^. The CLN6-affected subjects develop symptoms between 18 months and 8 years of age, and among the first clinical signs are ataxia, seizures, and progressive mental deterioration^10,11^.

Early diagnosis of CLN6 is essential for developing treatment and managing disease prognosis^10,12^. However, diagnosis is based on combined clinical symptoms and genetic testing and is often made at an advanced disease stage, which brings an unfavorable prognosis. Research on CLN6 has intensified over the past decade as extensive attempts were made to develop therapies and understand the disease pathology^12–14^. Even so, the disease mechanism underlying CLN6 pathogenesis remains unclear, and the development of an appropriate treatment is pending. Among the clinical needs awaiting to be achieved is the discovery of specific biomarkers for disease screening, prognosis, and monitoring^15,16^.

Liquid chromatography-mass spectrometry (LC–MS) is a robust platform that may uncover intricate metabolic pathways, deepen our understanding of the biochemical processes, and aid in biomarkers discovery in clinical and translational research^17,18^. Likewise, non-targeted metabolite profiling is a valuable tool for comparing metabolic changes between pathological and non-affected subjects. These together may assist in identifying a wide range of critical metabolites whose changes are linked to specific diseases^19,20^.

Only a few LC–MS-based metabolomics studies in NCLs have been conducted to identify potential biomarkers of disease progression^21,22^. Furthermore, CLN6 disease metabolomics studies are scarce and mainly related to animal models^23,24^. Hence, this implies that the metabolome of human CLN6 disease in neuronal-like cells is largely unexplored.

Here, we sought to compare the metabolomic profile in neuronal-like cells generated from fibroblasts of CLN6-affected and unaffected subjects (wild type). First, we set out to differentiate human CLN6 fibroblast lines into chemical-induced neuronal progenitor cells (ciNPC). Next, this model was used for biomarker discovery, which may aid in a rapid and inexpensive diagnosis and prognosis of the neurodegenerative disease^25^.

Considering that no human CLN6 studies have been published that addressed the metabolomic changes in cell lines of CLN6 patients, we aimed to analyze and compare the changes in the global metabolome of the induced neuronal-like cell lines from subjects with CLN6 disease to that of healthy subjects. The present cell-based study employed an untargeted LC–MS approach that, combined with in-depth data analysis, helped identify metabolic alterations linked to CLN6 disease.

## Results

The study was based on cell lines derived from subjects with CLN6 disease (carrying bi-allelic pathogenic variants, n = 3) and controls (wild type, n = 3). Inclusion criteria for the CLN6 subject were: (1) diagnosis of CLN6 based on genetic analysis, (2) both male and female subjects, (3) under 18 years at the time of sampling, and (4) unrelated individuals. The only difference in the inclusion criteria for control subjects was that they must not be diagnosed with any NCL disease and should not have any pathogenic or potentially harmful genetic variations. Detailed information about the included subjects is provided in Table 1.

**Table 1:** Clinical and genetic characteristics of subjects used in this study.

Group	Cell line	Sex	^a^Age at onset| sampling	^b^cDNA protein	Predicted effect	Type on DNA	Coding effect	Clinical significance	Evidence ACMG	Clinical symptoms
CLN6	I	M	5|16	c.-158_83del p.?	Deletion	Gross deletion	Effect unknown	Likely pathogenic	PVS1_S, PM2_P, PM3_P	Movement disorders, epilepsy, cognitive impairment
	II	F	5|5½	c.896C>T p.Pro299Leu	Missense	Substitution	Probably damaging	Pathogenic	PP3, PS3_P, PM2_P, PM3_S	Behavioural abnormalities, weight loss, cognitive regression, pyramidal and extrapyramidal signs, myoclonus, focal motor seizures
	III	F	4|5	c.794_796del p.Ser265del	In-frame	Deletion	In-frame	Likely pathogenic	PM2_P, PP1, PM3, PM4	Movement disorders, epilepsy, cognitive impairment
WT	I	M	–|8	NA						
	II	M	–|1	NA						
	III	F	–|11	NA						

WT wild type/control, ^a^year, ^b^homozygous, NA not applicable.

### Generation of neuronal progenitor cells (NPCs) from human dermal fibroblasts

**Figure 1:**
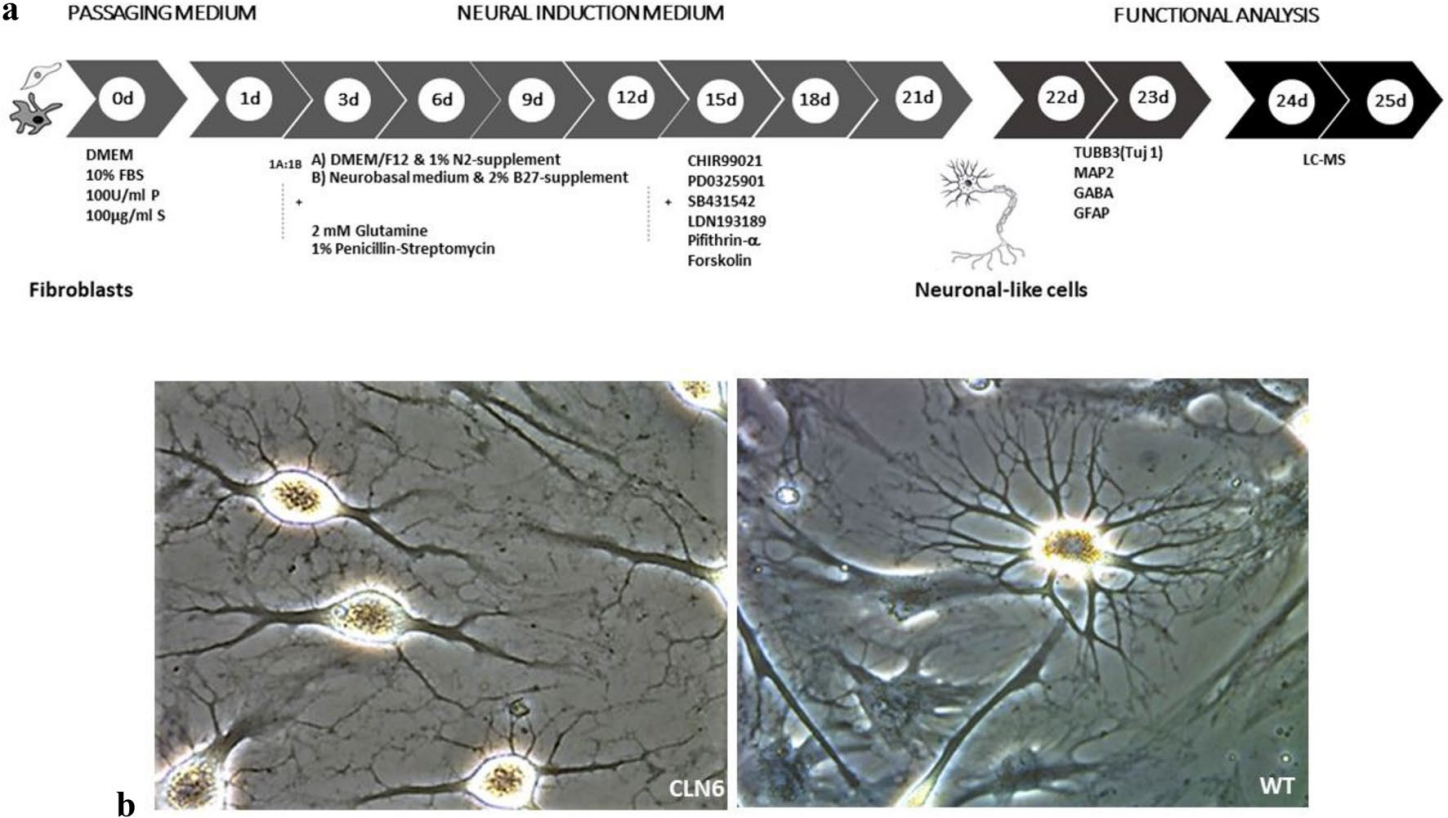
**(a)** Scheme of direct conversion of human dermal fibroblasts into neuronal-like cells. The experiment starts by plating the fibroblasts in the DMEM medium, which was designated as "Day”0." After 1 day, the cells were transferred to an induction medium containing chemical compounds and supplementary chemicals to promote differentiation into neuronal progenitor cells. (**b**) Representative microscope images of human ciNPCs morphologies at day 20 of development in the induction medium: (**a**) CLN6 group and (**b**) wild type/control group.

**Figure 2:**
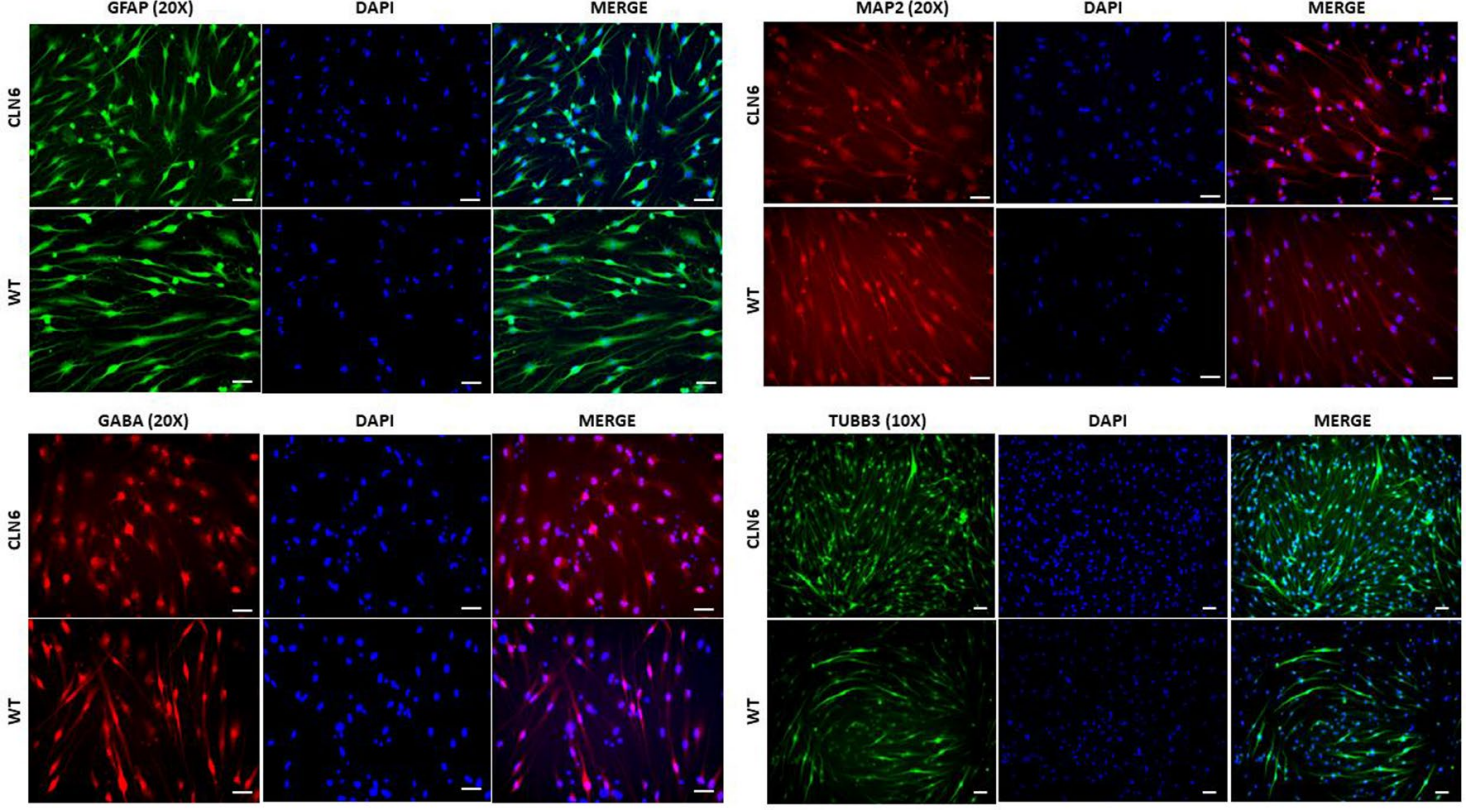
Immunofluorescence staining of neuronal cultures with anti-MAP2 antibody (mature neurons), anti-GABA (GABAergic neurons), anti-TUB βIII (immature neurons), and anti-GFAP antibody (astrocytes). Nuclei were stained with DAPI (blue). Scale bars = 50 μm. MAP, microtubule-associated protein 2; GFAP, glial fibrillary acidic protein; DAPI, 49,6-diamidino-2-phenylindole.

We used a simple and previously reported technique introduced by Dai et al*.* to directly generate ciNPCs from fibroblasts^26^. Figure 1a visually presents the sequential stages of the differentiation process using a cocktail of six small molecules, as outlined in the aforementioned protocol. As early as day 9 of differentiation, neuronal-like cell clusters were observed, and the small colonies of ciNPCs were depicted by day 20 post-induction (Fig. 1b). Three weeks after differentiation, the cells were harvested and checked for NPC markers via immunofluorescence staining (GABA, GFAP, TUJ1, and MAP2) and the metabolites extracted for LC–MS analysis. The differentiation resulted in the expression of neuronal markers, such as GABA (GABAergic neuron marker), GFAP (glial fibrillary acidic protein), TUJ 1 (Neuron-specific class III beta-tubulin), and MAP-2 (Microtubule-associated protein 2), as confirmed by immunofluorescence (Fig. 2). The CLN6 and WT NPCs had similar expression levels of the neuronal markers listed above. Immunofluorescence images revealed that ciNPCs express neuronal markers at day 20 of neuronal differentiation.

### Metabolomics analysis reveals distinct metabolic profiling in CLN6 subjects

Global metabolomic profiling of fibroblast-derived ciNPCs from CLN6 and healthy subjects was performed using a non-targeted mass spectrometry (MS) approach. After conducting the sample quality check, two replicates from each group [CLN6-1 (b3-t1, b3-t2) and control-3 (b2-t1; b2-t2)] were excluded from the batch. The exclusion was necessary because there was no signal in the total ion chromatogram, likely caused by sample evaporation prior to the LC–MS measurement. As a result, 16 replicates (data points) per group were available for further data analysis.

There were 2720 spectral features detected (Supplementary Table 1) and defined as molecular entities with a unique retention time (RT) and mass value (*m/z*). The coefficient of variation (CV) across all cell lines and cohorts was under 30% for more than 64% of the variables, meaning that both groups have a similar degree of variability in their concentration across all cell lines and cohorts.

**Figure 3:**
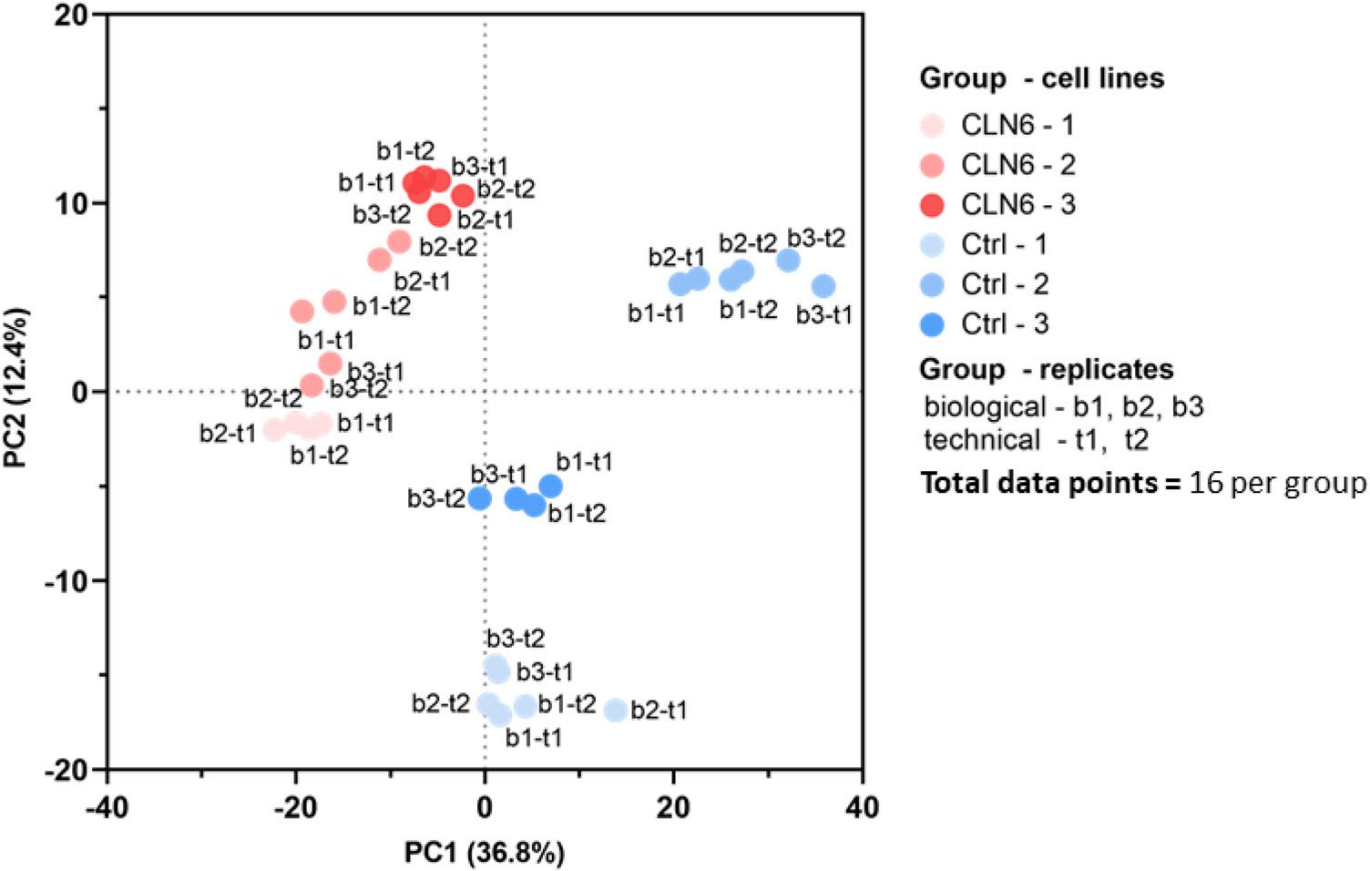
Principal component analysis (PCA) score plot of metabolites shows a separation of the CLN6 group from the control group, based on PC1 and PC2. The analysis was conducted using 16 data points (technical replicates) per group.

The datasets were then subjected to univariate and multivariate data analysis to assess the spectral features’ alteration in the two groups. Based on the entire metabolome datasets, we generated a principal component analysis (PCA) score plot that revealed a significant separation between CLN6 and the control group. As depicted in Fig. 3, PC1 contains the metabolites responsible for most between-group variations (36.8%), followed by PC2 (12.4%). As indicated by their high loadings in PC1, the glycerophospholipids such as PG 40:6, PG 40:7, PG 34:2, PG 32:1, and PG 40:4 contributed significantly to the overall variance captured by PC1.

**Figure 4:**
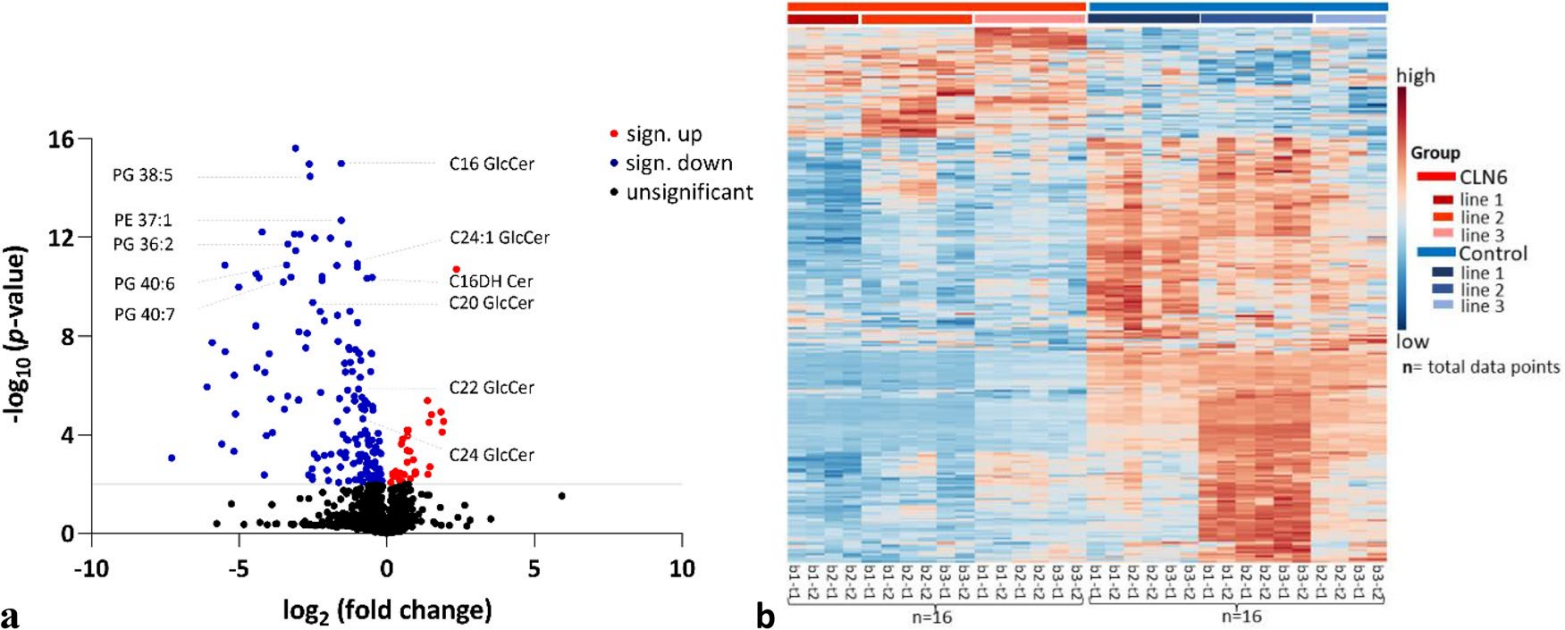
(**a**) Volcano plot displays the metabolites with a significant threshold of at least 2 × difference. The values are log-transformed. The threshold is shown as a grey line. The metabolites highlighted in red are upregulated for the CLN6 group, in blue are downregulated, and in black are not significantly changed (*p* ≤ 0.05). (**b**) Heatmap showing the abundance levels of the 10% most distinct metabolites screened across the two groups. The colors indicate increased (red) and decreased (blue) abundance for each metabolite across the samples. The letters (bottom) represent the biological replicas (b) and the technical replicas (t). Total number of replicates (data points) per group (n) = 16. Observation: most of the metabolites are decreased in CLN6 samples.

The individual selection of discriminating metabolites associated with CLN6 disease was based on folds change of at least two and *p* ≤ 0.05. Figure 4a displays the volcano plot depicting the fold changes in metabolites’ abundance and highlights the most significantly altered metabolites in terms of fold change and discriminatory power between the two groups. This analysis used the comprehensive dataset from Supplementary Table 1, incorporating the raw data obtained through LC–MS. The compounds chosen for further analysis were limited to the top 20 from the list of upregulated and downregulated compounds shown in the volcano plot. They were selected based on their intensity and discriminative power to differentiate between groups. It should be emphasized that the selected compounds were exclusively downregulated upon this features screening.

The metabolites were putatively annotated, which involved matching their mass-to-charge ratio (*m/z*) and retention time (RT) values to internal and external databases. Supplementary Table 2 lists the fold changes and Student’s t-test *p*-values (not adjusted) for these metabolites. Additionally, the metabolites with a fold change threshold of at least two are shown in a heatmap colored based on the actual peak intensity values to delineate the differences between the two groups (Fig. 4b, Supplementary Table 3).

**Figure 5:**
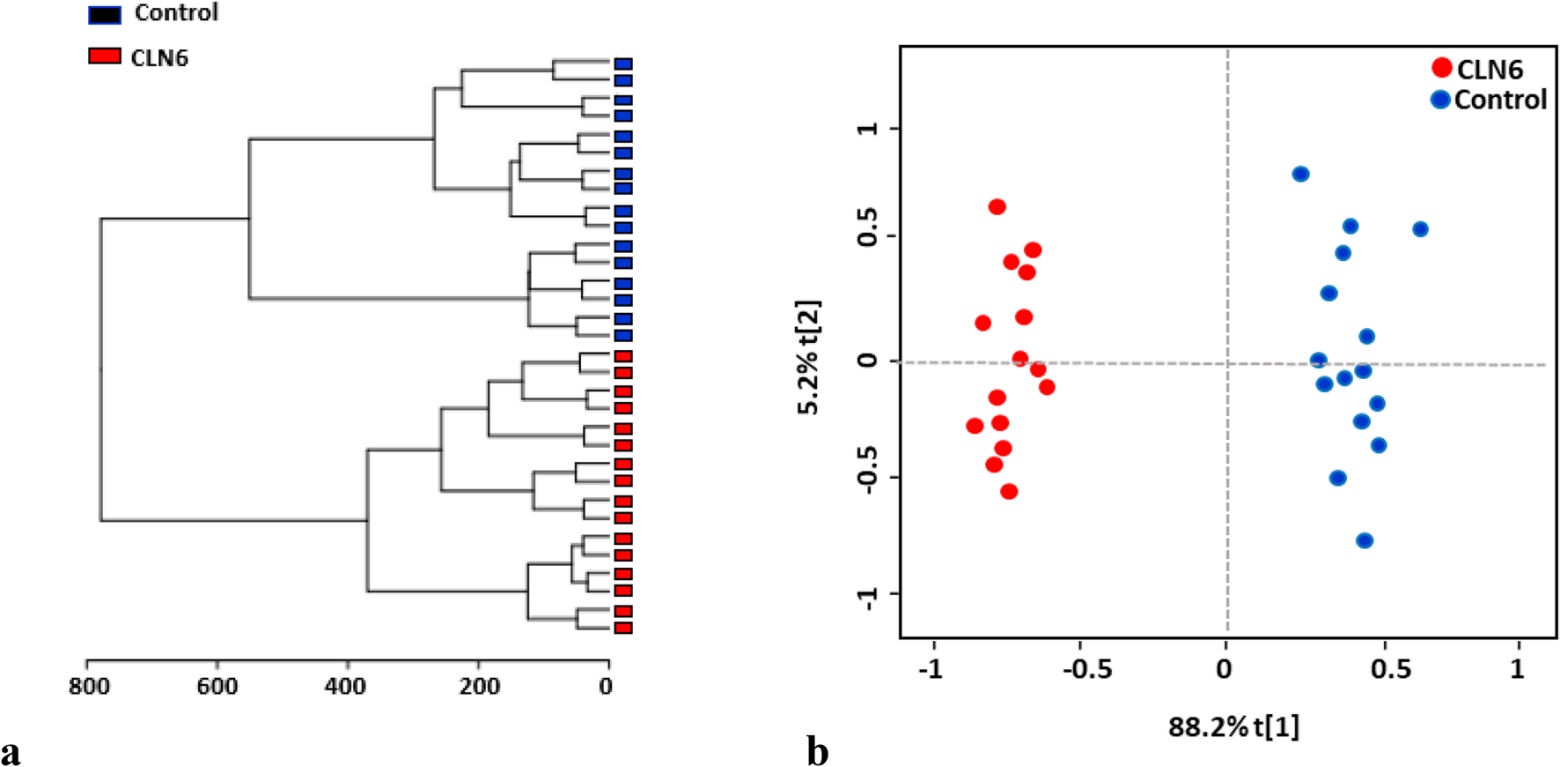
(**a**) Hierarchical clustering (Euclidean distance, Ward's clustering algorithm) confirms the biochemical differences between the two groups. (**b**) Ortho partial least squares-discriminant analysis (OPLS-DA) score plot from CLN6 and control. The clear separation between the two groups indicates that their metabolomic profile is distinct.

Two additional multivariate analyses were conducted to investigate the differences between the CLN6 and control subjects: unsupervised hierarchical cluster analysis (HCA) and ortho partial least squares-discriminant analysis (OPLS-DA). The unsupervised hierarchical clustering analysis based on the HCA technique was performed to group the data into clusters (Fig. 5a). The OPLS-DA analysis, on the other hand, was carried out to differentiate between the two cohorts and identify CLN6 dysregulated metabolites (Fig. 5b).

**Figure 6:**
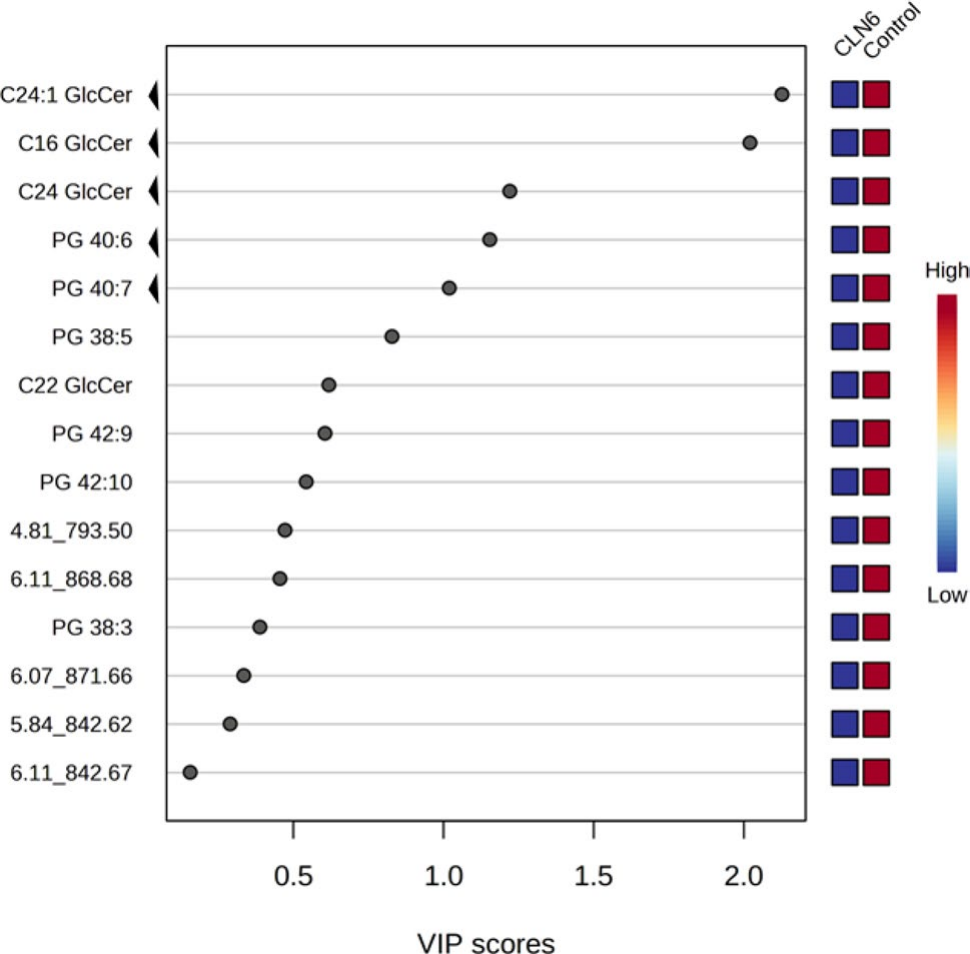
Variable importance in projection (VIP) score plot displays the metabolites with discriminating power derived from the PLS-DA model. Only the metabolites with the strongest discriminating power are represented here. These metabolites have contributed most to the variance between CLN6 and the control group. The non-annotated metabolites were named based on the retention time (rt) and *m/z* values; ‘arrows’ indicate the five most discriminating metabolites.

The metabolites with the highest discriminating power were chosen according to the Variable Importance in Projection (VIP). A VIP score ≥ 1.00 from PLS-DA was considered significant. Fifteen metabolites, all downregulated in the CLN6 group, were differentially expressed in the CLN6 with significant discriminative power from the control group (Fig. 6, Supplementary Table 2).

**Figure 7:**
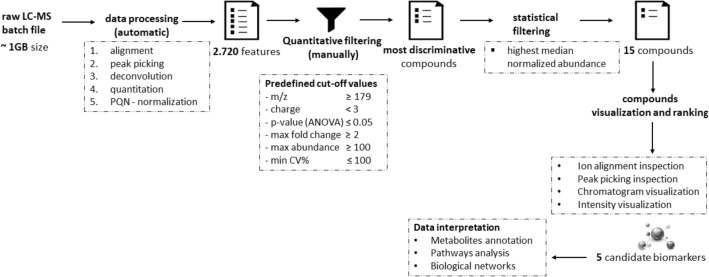
Metabolomics data analysis scheme for biomarker identification.

Five of all metabolites analyzed, met the stringent quality and quantity criteria in our detection process, as depicted in Fig. 7. These criteria entail a significant difference between the control and targeted disease group, a minimum fold change of twofold, and a high median normalized abundance. Furthermore, the compounds were ranked based on ion alignment, peak picking inspection, chromatogram, and intensity visualization. Consequently, these metabolites have been considered eligible in terms of quality and quantity for downstream characterization. The identified metabolites, likely biologically relevant within CLN6 disease, belong to the glycerophosphoglycerols and glycosphingolipids class. Specifically, they are represented by phosphatidylglycerols PG (40:7) and PG (40:6), as well as glucosylceramides C16 GlcCer, C24 GlcCer, and C24:1 GlcCer. The key characteristics covered by these metabolites are listed in Table 2.

**Table 2:** Characteristics of the top five putatively annotated metabolites.

Class	Synonim	Mass (*m/z,* n)	CCS (Å)	RT (min)	*P* value	Fold change	Adducts	Formula
Glycerophosphoglycerols	PG 40:7	819.5* m/z*	294.9	4.88	1.02E−10	-8	M−H	C_46_H_77_O_10_P
	PG 40:6	822.5n	299.1	4.97	2.51E−11	-3	M−H, M+Cl	C_46_H_79_O_10_P
Glycosphingolipids	C16 GlcCer	699.6n	285.0	5.31	1.39E−15	-3	M−H, M+Cl, [M+HCOO]-	C_40_H_77_NO_8_
	C24 GlcCer	811.7n	309.0	6.45	4.72E−06	-2	M−H, M+Cl	C_48_H_93_NO_8_
	C24:1 GlcCer	809.7 n	316.0	6.09	1.84E−13	-2	M−H, M+Cl, [M+HCOO]-	C_48_H_91_NO_8_

**Figure 8:**
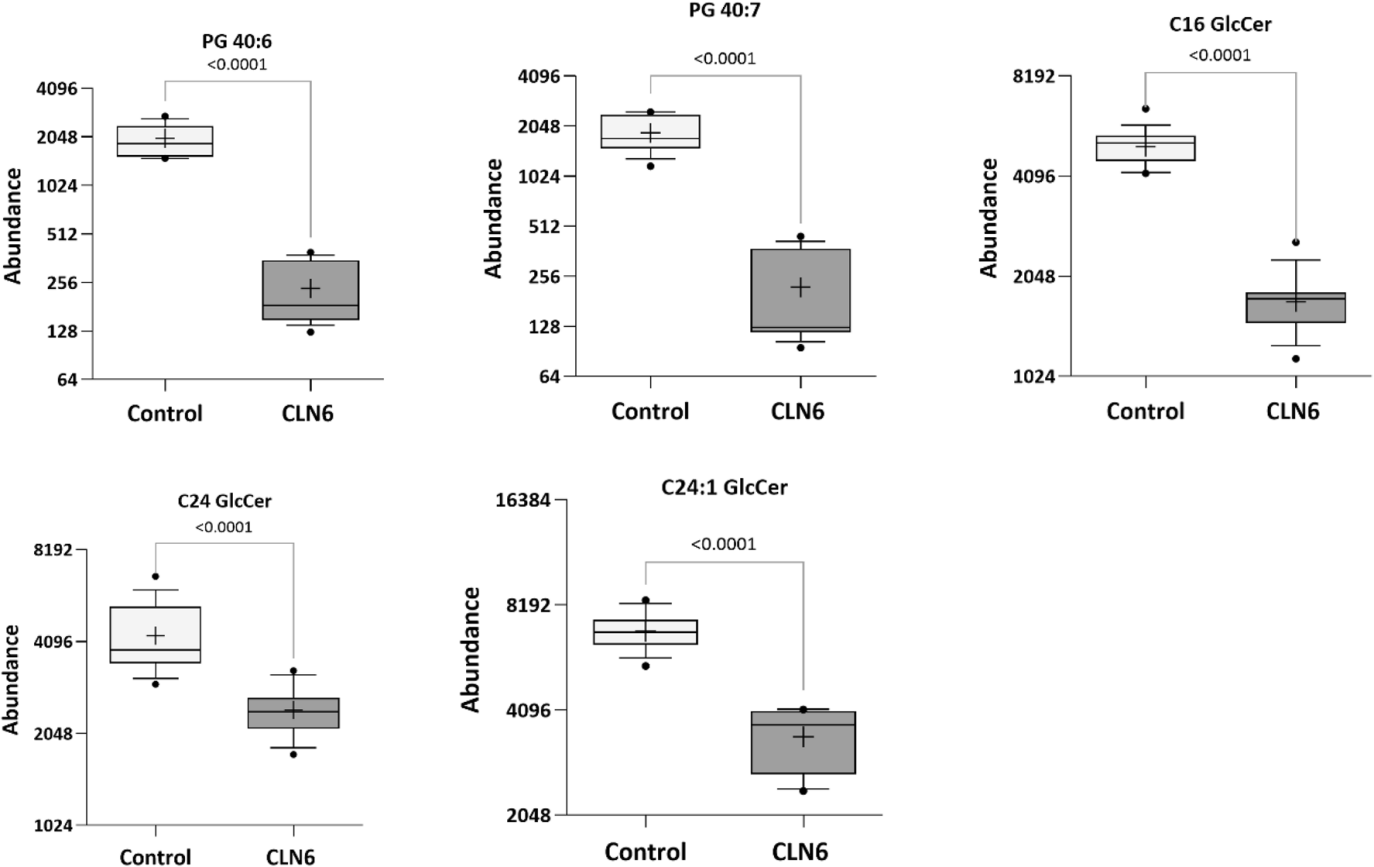
Potential metabolomic biomarkers for diagnosis of CLN6 disease. Representative Box plots showing the intensity of the top five metabolites in the two groups of neuronal-like cells [cell lines (n = 3) × biological replicas (n = 2–3) × technical replicas (n = 2)]. Whisker’s end = the 10th and 90th percentile, bars = min and max values, horizontal line in the boxes = median value, ‘+’ = mean. Dots represent the outliers.

They were selected as potential candidate biomarkers due to their differentiating power between the CLN6 disease and control groups (Fig. 8).

**Figure 9:**
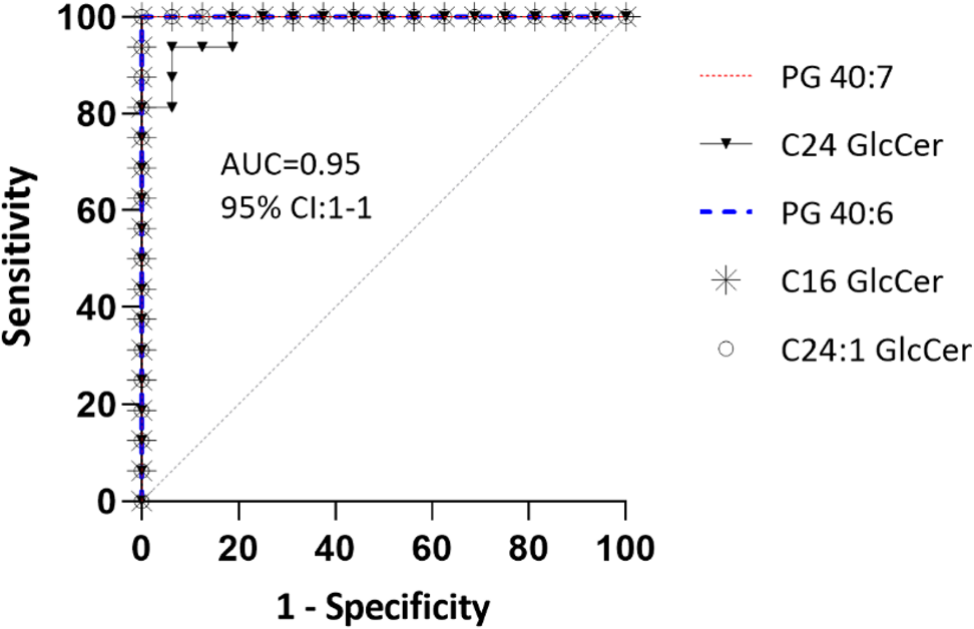
The overlaid ROC (receiver operating characteristic) curves were used to visualize the metabolites with the maximum sensitivity for differentiating CLN6 disease subjects from controls. Four compounds had 100% overlapping ROC curves.

The five metabolites’ overlaid receiver operating characteristic (ROC) curves reveal that these compounds delineate between the two groups with high precision and accuracy, indicating that they could be potential biomarker candidates for the early disease prognosis of CLN6 disease (Fig. 9).

### Pathway and network analysis of the differential expressed metabolites

**Figure 10:**
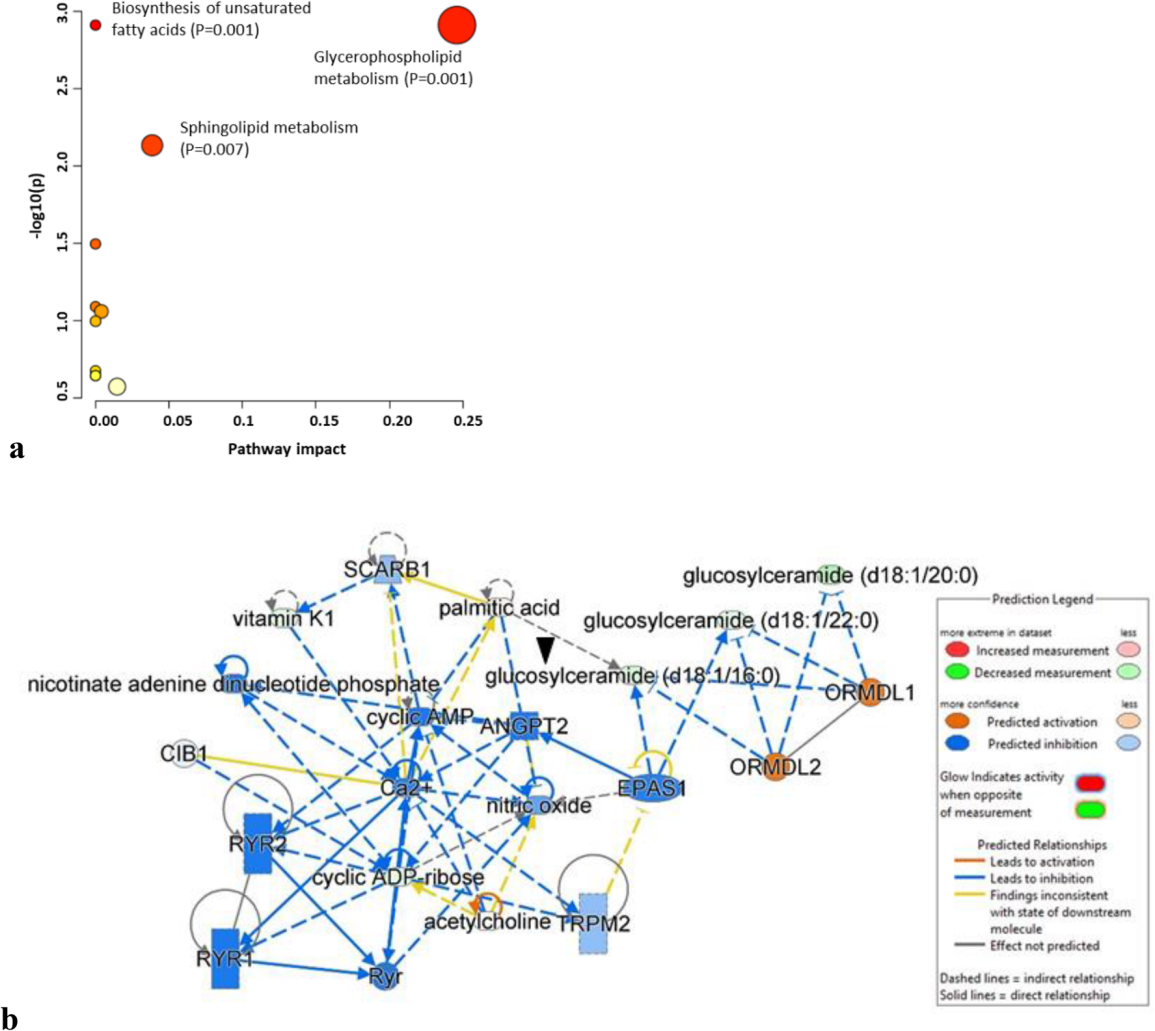
(**a**) The affected pathway and (**b**) pathway analysis in CLN6 patient samples as determined by MetaboAnalyst and Ingenuity Pathway Analysis software. The analysis reveals significant alterations in sphingolipid and glycerophospholipid metabolism and provides a network visualization of the interactions between different metabolic pathways and networks. The ‘arrow’ points to one of the metabolites among the five differentially expressed that is interconnected within the network.

To investigate the biological pathways and networks involved in the pathogenesis of CLN6 disease, we performed metabolomics data enrichment on selected metabolites using two software tools: MetaboAnalyst *v.* 5.0^27^ and Ingenuity Pathway Analysis (IPA) (QIAGEN Inc., https://digitalinsights.qiagen.com/IPA)^28^. The pathway analysis module in the MetaboAnalyst software illustrates the most affected metabolic pathways of the screened metabolites, as shown in Fig. 10a, with the sphingolipid and glycerophospholipid metabolism pathways being the most significantly altered. Additionally, we utilized IPA to generate networks that allowed us to further explore and understand the biological networks involved in disease pathogenesis. The IPA analysis on differentiated neuronal-like cells from CLN6 patients was performed with 298 mapped metabolites, of which 158 were downregulated, and 140 were upregulated. Among them, 39 molecules showed significant regulation when applying a cut-off of ± 1.5-fold changes and a p-value ≤ 0.05 (28 downregulated and 11 upregulated). The list of mapped molecules by IPA can be found in Supplementary Table 4, while a summary of the obtained results is presented in Table 3.

**Table 3:** Summary of IPA analysis.

Canonical pathway	p-value	Ratio		
NAD signaling pathway	4.87E−02	0.0526		

Master regulators	p-value	Target molecules in dataset	Activation	Network bias-corrected p-value
ORMDL2	6.33E−05	Glucosylceramide (d18:1/16:0) glucosylceramide (d18:1/20:0) glucosylceramide (d18:1/22:0)	1732	4.00E−04
ORMDL1	8.08E−05	glucosylceramide (d18:1/16:0) glucosylceramide (d18:1/20:0) glucosylceramide (d18:1/22:0)	1732	4.00E−04
EPAS1	2.54E−03	Glucosylceramide (d18:1/16:0) glucosylceramide (d18:1/22:0)	− 1414	2.00E−04
SCARB1	3.02E−02	Vitamin K1	− 1	1.52E−02
ANGPT2	2.52E−02	Cyclic ADP-ribose	− 1	6.60E−03
CIB1	5.05E−03	Cyclic ADP-ribose	− 1	6.60E−03
acetylcholine	2.02E−02	Cyclic ADP-ribose	− 1	6.60E−03

Functions annotation	Molecules	p-value		
Quantity of ceramide	Ca^2+^, EPAS1, GBA1, ORMDL1, ORMDL2, palmitic acid, PSAP, SCARB1	1.25E−14		
Quantity of Ca^2+^	Acetylcholine, ANGPT2, Ca^2+^, CIB1, cyclic ADP-ribose, cyclic AMP, nicotinate adenine dinucleotide phosphate, nitric oxide, palmitic acid, Ryr, RYR1, RYR2, vitamin K1	5.91E−14		
Release of Ca^2+^	Ca^2+^, cyclic ADP-ribose, cyclic AMP, nicotinate adenine dinucleotide phosphate, nitric oxide, Ryr, RYR1, RYR2, TRPM2	5.11E−11		
Mobilization of Ca^2+^	Acetylcholine, Ca^2+^, cyclic ADP-ribose, nicotinate adenine dinucleotide phosphate, palmitic acid, Ryr, SCARB1	8.53E−07		
Synthesis of lipid	Acetylcholine, ANGPT2, Ca^2+^, cyclic AMP, GBA1, nitric oxide, ORMDL1, ORMDL2, palmitic acid, SCARB1	2.37E−08		
Accumulation of nitric oxide	Cyclic ADP-ribose, EPAS1	1.56E−05		
Propagation of signaling of Ca^2+^	Cyclic ADP-ribose	2.61E−03		

The NAD signaling pathway showed significant association with the differentially expressed metabolites (p-value = 0.048, 5.3% overlap), and the deregulation of cyclic ADP-ribose (fold change = − 1.56, p-value = 0.00025). A regulator effect network was also identified (score 15) with associated cell signaling, molecular transport, vitamin, and mineral metabolism functions (Fig. 10b). Causal relationships were connected to the downregulated glucosylceramides via the upstream *ORMDL1* (p-value 8.08E−05) and *ORMDL2* (6.33E−05) sphingolipid biosynthesis master regulators linked to annotated functions such as the quantity of ceramide (p-value 1.25E−14), and synthesis of lipid (p-value 2.73E−08). Furthermore, the master regulator *EPAS1,* a transcription regulator often known as *HIF2A*, was also identified by IPA (p-value 2.54E−03, z-score − 1.41) in participating to the accumulation of nitric oxide (p-value 1.56E−05) both via the downregulation of glucosylceramides and cyclic ADP-ribose leading to the regulation of functions annotation like propagation of signaling of Ca^2+^ (p-value 0.00261) with the predicted downregulation of quantity of Ca^2+^ (p-value 5.91E−14), mobilization of Ca^2+^ (8.53E−07), and release of Ca^2+^ (p-value 5.11E−11). The participation of several upstream regulators, including the transporter *SCARB1* (p-value 0.0302), targeting the downregulated vitamin k1 in our dataset, the calcium and integrin binding 1 gene (*CIB1*, p-value 0.005), and the angiopoietin-2 protein coding gene (p-value 0.0252) have been identified by IPA as regulators of calcium homeostasis functions.

## Discussion

To our knowledge, this study is the first to use neuronal progenitor-like cells differentiated from human CLN6 fibroblast lines to identify differentiating metabolites that can distinguish CLN6 disease from the healthy control group.

Research on CLN6 disease aiming to understand the disease pathophysiology and develop therapies has expanded significantly over the past decade. However, according to a literature review survey on the PubMed^®^ database (https://pubmed.ncbi.nlm.nih.gov)^29^, few studies aimed explicitly at metabolomics investigation of CLN6 disease, and the ones reported were model organisms-based using, for example, sheep and mouse models^23,24^. Nevertheless, these used GC–MS and NMR platforms and discovered an alteration of the glutamine-glutamate metabolism and a decrease of GABA in their quest for altered metabolic pathways that lead to neuronal degeneration.

Given that no human CLN6 studies have been published that addressed the metabolomic changes in the cell lines of CLN6 patients, we aimed to analyze and compare the changes in the global metabolome of the induced neuronal-like cell lines from humans with CLN6 disease to those of healthy subjects. For this, an LC–MS metabolomics approach was employed to identify the metabolic pathways altered in CLN6 subjects and acquire further insights into potential markers of disease pathogenesis. The metabolic profiling analyses were performed on induced neuronal-like cell lines from subjects with CLN6 disease and human controls. Fifteen metabolites were markedly downregulated in CLN6 subjects and showed a robust discriminatory power between the CLN6 and the control group.

Results of pathway identification performed with the MetaboAnalyst *v* 5.0 software and IPA analysis revealed alterations in molecules relevant to sphingolipid and glycerophospholipid metabolism. Although no research has yet addressed the role of sphingolipids in the CLN6 disease, our findings imply that disruptions in sphingolipid metabolism are a feature of the CLN6 disease, which could represent a source for future biomarker discovery. Among the dysregulated metabolites related to CLN6 disease were five distinct metabolites with a VIP score greater than 1. They were represented by the two glycerophospholipids, PG 40:6 and PG 40:7, and the three sphingolipids, C16 GlcCer, C24 GlcCer, and C24:1 GlcCer.

Studies on Alzheimer’s disease subjects proved that altered glycerophospholipids levels might lead to neuronal damage, neuroinflammation^30,31^, and neurodegeneration^32,33^, features that resemble the NCL disorder^3^. Additionally, glycerophospholipids were proposed as putative biomarkers in neurodegenerative diseases^34^. Besides glycerophospholipids, dysregulated glucosylceramide levels have also been linked with neurodegeneration in lysosomal storage disorders (LSDs)^35^. Other investigations have shown a connection between increased glucosylceramide levels, neuroinflammation, and neuronal loss^36^.

Similarly, our study identified two distinct glycerophospholipids components (PG 40:6 and PG 40:7) with statistically low abundance in the CLN6 group. Moreover, the compounds with the most discriminatory power between CLN6 and the control group were C24:1 GlcCer and C16 GlcCer, highlighting their distinctive importance in the dysregulation of the sphingolipid metabolic pathways for CLN6 disease. However, in contrast to the studies mentioned above, where the levels were elevated, our results showed that glucosylceramides were downregulated in CLN6 compared to the healthy control group. Equivalent results were reported in a CLN9-deficient cells-based study, where diminished levels of ceramide, glucosylceramide, and other sphingolipids components were observed^37^.

Concerning the NCLs group, perturbed sphingolipid metabolism was described in various NCLs forms. One of the first studies that mentioned dysregulation in phospholipid metabolism was done on CLN1 and CLN3 disease patients^38^. Later investigations considered that the perturbed sphingolipid metabolism may represent a link between some forms of NCLs^39^. According to the study conducted in CLN3-defective cells, a decrease in various sphingolipids, such as lactosylceramides and glycosphingolipids, and up to a 60% reduction in the level of various HexCer components as compared to the control group was revealed^40^. A more recent study on CLN5 disease reported a similar outcome by exploring the connection between CLN5 disease and the degradation of sphingolipid metabolism^41^. These findings infer that CLN3 and CLN5 play a critical role in the changes in sphingolipid metabolism. Altogether, several studies on infantile and late infantile variants mentioned abnormal lipid metabolism^3^, and others noted changes in the composition of various phospholipid and ceramides classes^38,42,43^ (Table 4), yet our work is the first to link the sphingolipid metabolism to CLN6. Furthermore, the role of calcium signaling has been investigated in several models showing an important role of calcium homeostasis in NCL pathology^44,45^ and elevated calcium-binding protein calbindin 1 (CALB1) levels in cerebrospinal fluid from CLN2 and CLN3 disease patients^46^. Similarly, our study found that the downregulation of glucosylceramides, cyclic ADP-ribose, and vitamin K1 was associated with the inhibition of calcium signaling in CLN6 disease.

**Table 4:** List of lipid species involved in dysregulated lipid metabolism in NCL disease.

NCL type	Lipid class	Lipid species	Level	Refs
CLN1	Phospholipids	LBPA (36:2; 34:1); PC (32:1, 34:1)	High	^37–39,42^
		PC (32:1, 34:1); PI (36:3, 38:5, 38:3); PC (32:1, 34:1)		
		PE (38:5, 36:4, 38:5, 36:2, 34:1, 34:2)		
		PS (34:4, 38:3, 40:3, 34:1, 36:1, 36:2, 38:2)		
		LBPA (38:1); PC (38:4, 38:6)	Low	
		PE (40: 6, 38:4, 40:4); PI (38:4, 40:6); PS (40:6)		
	Sphingolipids	SM (16:0, 16:1, 18:1)	High	^38^
		SM (20:0)	Low	
CLN2	Phospholipids	GPE (18:1)	High	^43^
		GPE (16:0, 18:0)	Low	
CLN3	Phospholipids	LPE (20:3); PC (32:1, 34:1, 36:3)	High	^37,40^
		PS (38:3); PI (38:3); PE (38:3, 40:3)		
		PA (36:1, 36:2); PC (38:4); LPI (18:0, 20:4); LPC (20:4)	Low	
		PG (34:2, 34:1); PE (40:4); PI (36:1, 36:2, 36:3, 36:4, 36:5)		
	Sphingolipids	Cer (16:0, 24:0, 24:1); SM (24:1)	High	^39,40^
		GM3 (d18:1/24:1, d18:1/16:0, d18:1/24:0)		
		GM3 (d18:1/24:1, d18:1/16:0, d18:1/24:0)		
		GD1 (d18:1/25:0, d18:1/16:0, d18:1/22:0); SM (14:0, 15:0)	Low	
		HexCer (22:0, 20:0, 18:0, 23:0); LacCer (16:0, 24:0)		
	Sterols	CE (18:2, 18:3)	Low	^40^
CLN9	Sphingolipids	Cer (16:0, 24:0, 24:1)	Low	^37,39^
		dhCer (16:0, 24:0, 24:1)		

Cer: ceramide; CholE: cholesterol Ester; dhCer: dihydroceramides; GD1: monosialoganglioside D1; GM3: monosialoganglioside GM3; GPE: glycerophosphoethanolamine; HexCer: hexosylceramide; LacCer: lactosylceramide; LBPA: lysobisphosphatidic acid; LPC: lysophosphatidylcholine; LPE: lysophosphatidylethanolamine; LPI: lysophosphatidylinositol; PA: phosphatidic acid; PC: phosphatidylcholine; PE: phosphatidylethanolamine; PG: phosphatidylglycerol; PI: phosphatidylinositol; PS: phosphatidylserine; SM: sphingomyelin.

In summary, the current study compared the metabolomic profile of CLN6-neuronal progenitor-like cells derived from fibroblast to the control group. Our findings showed that five metabolites were significantly dysregulated in the cell lines from CLN6 subjects and may be considered potential candidate biomarkers for CLN6 disease. Additionally, the metabolic pathway analysis suggests the involvement of the sphingolipid, glycerophospholipid metabolic pathway, and calcium signaling in the mechanism behind the CLN6 disease progression, which is oriented toward the downregulation of sphingolipids and that of glycerophospholipids metabolism.

While our findings enhanced our understanding of the metabolomics of the CLN6 disease, scale-up research involving additional cell lines and diverse patient cohorts is needed to validate the observed pattern in our data. The corroboration of our findings would pave the way for advanced metabolomics studies of CLN6 disease that may uncover potential therapeutic targets of CLN6 disease.

Given the significant milestones achieved in our study, which underscore the potential of ciNPCs within an LC–MS-based metabolomics approach for biomarker discovery, we recognize the need to address the following key research directions: (i) expand and diversify the cohort to encompass a broader range of ages and genders to ensure a more comprehensive dataset and greater generalizability of our findings; (ii) incorporate additional NCL disease subtypes for comparative analysis thus assessing the specificity and relevance of potential biomarkers across diverse subtypes; (iii) undertake targeted MS/MS research, building upon the spectral features reported in this study to deepen our understanding of the involved metabolites and pathways, ultimately amplifying the precision and impact of our research.

The findings presented in this study offer valuable insights into the metabolic changes associated with CLN6 disease. However, further research is recommended to understand our results’ broader implications fully. To this end, we propose several directions for future investigations:

1. Incorporating cellular vitality and stability assessments into upcoming research to enhance metabolomics data precision and the reliability of biomarker discovery studies. While the primary goal of this study was to assess the feasibility of ciNPC cells for identifying CLN6 biomarkers through metabolomics analysis, it is crucial to consider the potential impact of cellular vitality and health on metabolomics profiling, potentially affecting the detection of subtle disease- or treatment-related effects. Therefore, future study endeavors should expand their scope to include an assessment of cellular health and stability, such as assessing DAPI+ spots to evaluate vitality and cell death rates and employing Western blot analysis to evaluate protein expression changes as well as quantitative staining. These methods provide valuable insights into ciNPCs, ultimately enhancing understanding of cellular pathophysiology, particularly within the context of biomarker discovery.
2. Validation studies involving fibroblast cells under non-induced conditions are essential to ensure the reliability of the identified metabolites in CLN6 disease. This additional validation step will offer valuable insights into the accuracy and specificity of the identified metabolites as potential biomarkers, both in cost-effective cells and in minimal-invasive specimens. Thereby, it will significantly enhance the potential of our research findings for future applications.
3. Conducting targeted experiments, such as mass spectrometry-based proteomics, to identify and validate the putative interactors and regulators predicted by in-silico analysis will yield robust evidence to support the functional significance in the context of CLN6 disease.

To conclude, this study is the first to examine the metabolome of human CLN6 ciNPCs, which provides metabolomics insight into the pathogenesis of CLN6 disease. Over 2700 spectral features were relatively quantified, two altered pathways were determined, and five putative biomarker candidates were identified. Altogether, this demonstrates a solid basis for the applicability of LC–MS-based metabolomics, which ultimately, could lead to an earlier diagnosis and prognosis of CLN6 disease and ease monitoring the effectiveness of upcoming therapeutic trials.

## Materials and methods

### Cell lines

The biobank “Cell Line and DNA Bank of Genetic Movement Disorders and Mitochondrial Diseases”, a member of the Telethon Network of Genetic Biobanks (project no. GTB12001), funded by Telethon Italy, and the EuroBioBank Network^47^ provided us with the CLN6 fibroblast lines [CLF064, CLF121, and CLF210]. The following cell lines (wild type) were obtained from the NIGMS Human Genetic Cell Repository at the Coriell Institute for Medical Research: [GM0839, GM0565, and GM0203].

### Chemicals and reagents

Ultra-high-performance liquid chromatography (UHPLC), grade acetonitrile (ACN), formic acid (FA), and methanol (MeOH) were purchased from Biosolve (Dieuze, France). Water LC–MS grade was purchased from VWR (Darmstadt, Germany). DMEM high-glucose medium (Gibco, Grand Island, NY), DMEM/F12 medium (12634010, Gibco), neurobasal medium (21103049), fetal bovine serum (FBS) (26140079), and 1% penicillin–streptomycin solution (15140122) were purchased from Thermo Fisher Scientific. Phosphate-buffered saline (10×) (PBS) (AM9624) was purchased from Invitrogen. B-27™ Supplement (50×) (10828010, Gibco), N2 Supplement (100×) (17502048, Gibco), and l-Glutamine (100×) (25030081, Gibco) were obtained from Life Technologies (Grand Island, New York, USA). CHIR99021 (130106539), SB431542 (131106275) were from Miltenyi Biotec (Teterow, Germany) and PD0325901 (PZ0162), LDN193189 (SML0559), Pifithrin-a (P4359) and Forskolin (F3917) were all from Sigma Aldrich (Taufkirchen, Germany).

### Cell culture

The fibroblast lines were maintained at 37 °C and 5% CO_2_ in high glucose Dulbecco’s modified Eagle medium (DMEM) (Gibco, Thermo Fisher Scientific, Waltham, MA, USA), supplemented with 10% fetal bovine serum (FBS) (Gibco, Thermo Fisher Scientific, Waltham, MA, USA), 100 U/mL penicillin, and 10 µg/ml streptomycin until they reached 90% confluence.

### Generation of chemical-induced neuronal progenitor cells

The direct chemical conversion of fibroblasts into neuronal-like cells was performed according to a previously published methodology by Dai et al.^26^. According to the protocol, once the cells reach the desired confluency, they are further switched into a neuronal medium made of a mixture of one-part DMEM/F12 (1% N2 supplement, Gibco) and neurobasal medium (2% B27 supplement, Gibco), and another part was a cocktail made of six chemicals (*v*1:* v*1). The chemicals known to aid in the NPC differentiation were represented by SB431542 (2 μM, TGF-β inhibitor), CHIR99021 (1 μM, GSK3b inhibitor), PD0325901 (1 μM, MAPK inhibitor), LDN193189 (1 μM, BMP inhibitor), Pifithrin-α (5 μM, p53 inhibitor), and Forskolin (7 μM, cAMP activator). The cells were cultured until day 21, and the medium changed every third day.

### Immunofluorescence

Cells were washed with PBS and fixed with 4% paraformaldehyde (Sigma-Aldrich, St. Louis, MO) for 15 min, then permeabilized in PBS containing 0.3% (w/v) Triton X-100 for 10 min, lastly blocked in PBS containing 4% (w/v) BSA for 2 h. Cells were incubated with the following primary antibodies (1:250 dilution) for 2 h at room temperature): anti-βIII-tub (Biolegend, cat. 801201), anti-MAP2ab (Merck Millipore, cat. AB5622), anti-GFAP (Biolegend, cat. 83721), and anti-GABA (Sigma Aldrich, cat. A2052). Subsequently, the cells were rinsed three times with 0.1% (w/v) BSA in PBS-Tr and incubated with the secondary antibody (1:500 dilution) for 1 h at room temperature in the dark (Alexa Fluor 488 A11029, and Alexa Fluor 568, A11036, Invitrogen). Nuclei were counterstained with DAPI (Invitrogen, cat. 1:10000) for 45 min. Cell images were acquired using a Keyence fluorescence microscope BZ-X710E equipped with the BZ-X800 Analyzer software (Keyence, Osaka, Japan) with a 20X Plan-Apo Gamma NA 0.75 objective and fluorescence filter set for GFP, TRITC, and DAPI.

### Sample preparation and metabolites extraction

While the adherent cell plates were kept on dry ice, the medium was removed, and the cells were quickly rinsed with 1 mL of 0.9% NaCl (4 °C) (Baxter, Sydney, Australia) to remove extracellular metabolites. A 600 µL extraction solvent (methanol: water, 3:1 *v/v*), prechilled in a − 80 °C freezer for at least 1 h, was added to the cells, and the cells detached using a scraper while the plates were kept on dry ice. The cell suspension was transferred into a 1.5 mL Eppendorf tube containing 10 µL internal standard prepared using 200 ng/mL Lyso-Gb2 (Matreya LLC, State College, PA, USA) dissolved in methanol. The mixture of cells, extraction solvent, and internal standard (IS) was vortexed vigorously. It was then centrifuged at 14,000×*g* for 20 min at 4 °C. A volume of 150 μL supernatant was transferred into an LC–MS glass vial. The quality control (QC) was generated by pooling 5 μL of each sample. Blank samples consisted of 100% LC–MS water. Before injecting the standard samples, blank and pooled samples were injected five times each in the beginning to establish system equilibrium. Throughout the batch, 5 µL of the pool, blanks, and standard samples were injected intermittently during the run to ensure the stability of the LC–MS system.

### Chromatographic and mass spectrometric conditions

Mass spectrometry was performed on a Waters^®^ i-Class ACQUITY UPLC (Waters, Borehamwood, UK) coupled to a Vion™ IMS Q-Tof™mass spectrometer (Waters, Borehamwood, UK) equipped with an ESI ion source, system operating in negative (ESI −) ionization mode. The LC–MS method was previously reported^48^. It was based on a 5 μL aliquot extract injected into a Kinetex EVO (C18, 2.1 × 150 mm, 5 μm) LC column (Phenomenex, Aschaffenburg, Germany) preheated to 50 °C at a flow rate of 0.5 mL/min. Analytes were eluted using a linear gradient ranging from 1 to 100% B (50 mM formic acid in methanol: acetonitrile 1:1 *v/v*) and A (50 mM formic acid in water). The following settings were used for mass spectrometric acquisition: High Definition MS^E^ (HDMS^E^), capillary voltage 1.2 kV, source temperature 150 °C, desolvation temperature 600 °C, desolvation gas 1000 L/h, cone gas 50 L/h, low collision energy 6 eV, high collision energy ramp 20–40 eV, scan mass 50–1000 m*/z*, scan time 0.5 s. Each signal had three identifiers: retention time in min (RT), ion mass (m/z), and CCS (collision cross-section). Leucine-enkephalin (Sigma-Aldrich, Taufkirchen, Germany) (1 ng/μL) was used as a lock mass reference compound *([M−H*]− = 554.2615, negative ion mode).

### Metabolomic data processing

The raw MS data were acquired using Unifi software v1.9 (Waters, Borehamwood, UK) and exported as Unify export packages (.uep). The generated datasets were imported to Progenesis QI software v 3 (Nonlinear Dynamics, Newcastle upon Tyne, UK) for automatic data processing. The following steps were part of the data processing and analysis workflow: retention time correction, experimental design setup, peak picking, probabilistic quotient normalization (PQN)^49^, deconvolution, and compound identification. The metabolites were individually assessed for statistical relevance and robustness. Only the variables that met the following quality filters were selected: significant difference between the control and CLN6 disease group (*p* ≤ 0.05), fold change at least twofold, charge ≤ 3, mass-to-charge ratio (*m/z*) ≥ 179, and a median normalized abundance ≥ 100 counts relative to the reference compound in at least one of the cohorts. The peak intensities of the selected compounds were transformed into .csv files and uploaded into the ‘Statistical Analysis’ toolbox of MetaboAnalyst v5.0 at http://www.metaboanalyst.ca^27^. Canonical pathway analysis was conducted using Ingenuity Pathway Analysis (IPA) software from QIAGEN (Ingenuity Systems, QIAGEN, Redwood City, CA, USA) with 298 mapped molecules by IPA using either Human Metabolome database (HMBD) or CAS registry number, or PubChem CIS IDs. A cut-off of ± 1.5-fold changes and p-value ≤ 0.05 was applied. The Euclidean distance metric and the 'Ward' clustering algorithm were used to create dendrograms. Heatmap with enforced sample grouping displayed value distributions and ranges.

### Metabolite database searching

Metabolites were identified based on monoisotopic mass, retention time, and collision cross-section. The obtained features were matched against several metabolite databases. Our in-house compound library, Human Metabolome Database^50^, PubChem^51^, ChemSpider^52^, and LIPID MAPS^®^ Structure Database (LMSD) were among the databases used in this study.

### Statistical analysis

Multivariate analysis of LC–MS data and pathway analysis were performed using the open-source software MetaboAnalyst 5.0^27^. The box-and-whisker plots, ROC, and volcano plots were generated using the GraphPad Prism (version 9.5.0) software (GraphPad Software, Inc., San Diego, CA, http://www.graphpad.com). A student’s t-test was applied to identify with a 95% confidence level and 5% false positive (false discovery rate, FDR). The level of significance was set at *p* ≤ 0.05.

### Supplementary Information

Supplementary Table 1.

Supplementary Table 2.

Supplementary Table 3.

Supplementary Table 4.

## Data Availability

The datasets generated and analyzed during the current study are available from the corresponding author upon reasonable request.
